# Thermal transport investigation and shear drag at solid–liquid interface of modified permeable radiative-SRID subject to Darcy–Forchheimer fluid flow composed by γ-nanomaterial

**DOI:** 10.1038/s41598-022-07045-2

**Published:** 2022-03-03

**Authors:** Adnan, Waqas Ashraf, Ilyas Khan, M. Andualem

**Affiliations:** 1 Department of Mathematics, Mohi-ud-Din Islamic University, Nerian Sharif AJ&K, 12080 Pakistan; 2 Department of Applied Mathematics and Statistics (AM&S), Institute of Space Technology (IST), Islamabad, 44000 Pakistan; 3 Department of Mathematics, College of Science Al-Zulfi, Majmaah University, Al-Majmaah, 11952 Saudi Arabia; 4 Department of Mathematics, Bonga University, Bonga, Ethiopia

**Keywords:** Engineering, Mathematics and computing, Nanoscience and technology

## Abstract

The modern world moves towards the art of nanotechnology which is impossible without the analysis of thermal performance and thermophysical featuring of nanofluids. Therefore, a case study for Darcy–Forchheimer Flow (DFF) (γ-Al_2_O_3_/H_2_O)nf over a permeable Stretching Rotating Inclined Disk (SRID) under the impacts of thermal radiation and viscus dissipation is organized. The nanofluid is synthesized by novel γ-aluminum nanomaterial and pure water. Then, the problem is formulated properly via similarity equations by inducing empirical correlations of (γ-Al_2_O_3_/H_2_O)nf with their thermophysical attributes. A numerical algorithm is successfully implemented for mathematical analysis and furnished the results for DFF of (γ-Al_2_O_3_/H_2_O)nf. It is inspected that the F_r_ opposes the motion and the fluid moves promptly by increasing the strength of stretching parameter. The temperature of (γ-Al_2_O_3_/H_2_O)nf enhances due to higher dissipation and fraction factor favors the thermophysical attributes of (γ-Al_2_O_3_/H_2_O)nf. Therefore, the nanofluid has high thermal performance rate and would be better for industrial and engineering purposes.

## Introduction

Heat transfer remains a critical problem of the researchers, engineers and industrialist from many decades. The solid reason behind this matter is the amount of heat transfer that required to accomplish various industrial products and for engineering purposes. Due to poor thermal performance of the regular liquids, industrialists and engineers are unable to acquire the desired demands of modern world. Therefore, a such class of fluids is needed which have high thermal transport characteristics and beneficial for industrial applications. The development in the modern world is impossible without the fluids having rich thermal attributes. Therefore, researchers and engineers in the fields supposed that the regular liquids could be made better for thermal transport by dispersing tiny particles of different metals. These particles dissolved in the regular liquids uniformly and thermally in equilibrium. Finally, a class is developed successfully which unlock the door towards innovative modern world. This class is named as “Nanofluids” and early significant contribution in this regard was made by Choi^1^.

After the development of nanofluids, researchers and scientists added the new inventions in the modern world era. The researchers paved their attentions on this significant class of liquids and made it more effective by studying it deeply. Therefore, new studies to be born regarding the investigation of heat transfer mechanism in the nanoliquid composed by various metals and different host liquids. Many studies regarding the heat transfer in the nanofluids under various geometries is reported so far. Among these, the inspection of nanoliquid dynamics between or over rotating disks attained much interest of the researchers and engineers. The reason of much popularity of such flows is their applications comprised in rotating disc reactor, oceans renewable power technologies and in other engineering system as well.

Recently, Kumar et al.^2^ organized the analysis of nanoliquid composed by CNTs. The fluid is bounded between two discs that placed parallel to each other. They studied the model subject to thermal radiation and porous medium effects. The problem was modeled properly via similarity equations and obtained dimensionless version comprising significant governing quantities. After careful analysis of the model, they examined that SWCNTs based nanoliquid has tendency to improve the local thermal performance and shear stresses than MWCNTs based-nanoliquid. The heat transport featuring in hybrid nanoliquid past a spinning disc under the influence of partial slip is examined by Li et al.^3^. They used hybrid nanoliquid composed by the additives of silver and MgO in the host liquid. For mathematical treatment of the model, they implemented fractional technique and plotted the results for the dynamics of the fluid against the governing quantities.

The investigation of second-law analysis is imperative and it has rich applications in aerodynamics, Fluid mechanics and in mechanical engineering. Therefore, entropy generation featuring in ferro-fluid by inducing the influences of electric field and Lorentz forces is organized and deeply discussed by Rout et al.^4^. They found that the heat transfer efficiency of the nanoliquid is improved by strengthen the surface convection and high fraction factor the nanomaterial also favors the thermal transportation. The study of heat transfers due to turning of disc is reported in Ref.^5^. They analyzed the heat transfer properties of different nanoliquids composed by various metals. Among the nanoliquids used, they reported that the nanoliquid comprising Cu particles is better conductor than rest of the nanoliquids used in the study.

Hydrothermal investigation in TiO_2_-GO nanoliquid between two disks subject to thermal radiation and Lorentz forces is conducted by Zangooee et al.^6^. They organized the model for stretchable disks by considering homogeneous/heterogeneous reactions and resistive heating. The model is then treated by implementing new mathematical technique known as AGM (Akbari Ganji Method) and provided the graphical results against the governing quantities. The augmentations in the nanoliquid temperature and Nusselt number are reported by increasing Re and stretching number, respectively. The behaviour of heat and mass transportation in the hybrid-nanoliquid over a disk oriented clock-wise is presented in Ref.^7^. The problem is organized for hybrid-nanoliquids containing ferrite and CNTs nanomaterials. The results for the dynamics are elaborated via graphs by implementing HAM technique. They reported that the liquid temperature and momentum is intensifies by increasing the disk rotation. Further, the used hybrid-nanoliquid is declared as better heat transport conductor than carrier liquid as for as thermal enhancement concerned.

The viscous dissipation contributed significantly in the dynamics of nanoliquids. Keeping in mind, Gul et al.^8^ arranged the analysis of nanoliquid past a turning disk. They contemplated the unsteady effects, Lorentz forces and viscous dissipation in the model. An enhanced temperature and velocity in the nanoliquid are observed by altering $\phi$ and $\omega$ while; increasing estimation of S opposes the nanoliquids motion. The paramount effects of Arrhenius energy and binary chemical reaction on the study of nanoliquid over a spinning disk is contributed by Asma et al.^9^. The model is organized via similarity equations and solved mathematical by implementing suitable technique. The results are plotted and a comprehensive discussion is provided in the study. Some significant studies concerning heat transfer in the nanofluid and hybrid-nanoliquid are reported in Refs.^10–12^ and the literature cited therian.

In the last few decades, the study of the composition of various metals, carbides, carbon nanotubes, ferromagnetic materials, Silver and nano diamond particles and base solvents like engine oil, kerosene oil (KSO), ethylene glycol (EG) and water etc. attained huge popularity in the modern world research communities. As, modern world could not move forward without the various sort of nanofluids, hybrid or ternary hybrid that playing significant role in the different industrial products, manufacturing of multiple vehicle parts, cooling systems and in aerodynamics. The integrated nanoparticles in the base solvents enhance the thermal conductivity of the resultant composition with superior heat transport characteristics (that lacks the conventional liquids) which allow them for industrial and engineering applications. In addition to these, different flow conditions like first and second order slip, thermal jump, convective heat condition and induction of magnetic field, Joule heating, heat generation/absorption, porosity effects and thermal radiations effects in the governing model are the key parameters in the analysis of above-mentioned heat transfer fluids. The latest vital studies in this regard are reported by different researchers^13–26^.

The significance of heat transport rate is unavoidable in the modern technological world because it is a primary need of industrialists and engineers. Form the above cited literature one thing is cleared that Fluid Dynamists are very curious about the study of heat transfer in conventional nanoliquid and hybrid nanoliquid synthesized from different base solvents and nanomaterials. Among them, Al_2_O_3_ and γ-Al_2_O_3_ became very famous due to their empirical correlations and their effects on the resultant nanoliquid. It is noted that heat transport in the nanoliquids composed by Al_2_O_3_ and γ-Al_2_O_3_ and base solvent in the presence of Darcy Forchheimer effects, thermal radiation, combine convection and viscous dissipation over a slippery SRID (Stretching Rotating Inclined Disk) is a huge research gap in the literature and not explored so far. To fill this massive research gap, a comprehensive study is organized to cover this gap and fulfill the expectation of the researchers and industrialists regarding the heat transport. In addition to heat transport, shear stresses, local Nusselt number and behaviour of empirical correlations versus volumetric fraction are the part of the study.

## Development of the model

### Model statement and geometry

**Figure 1:**
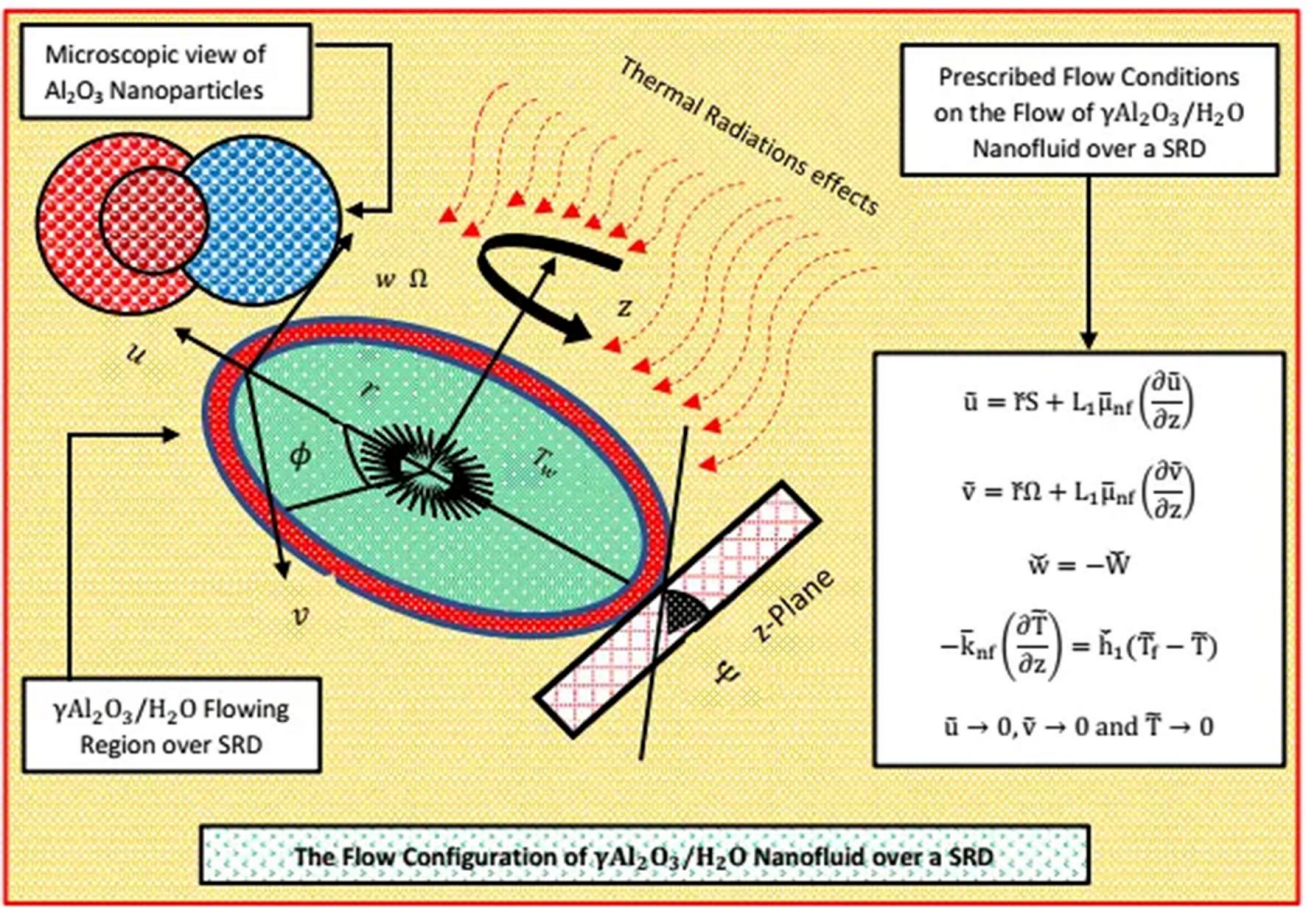
The Flow scenerio γ-Al_2_O_3_/H_2_O nanofluid.

The study is organized for steady DFF (Darcy–Forchheimer Flow) is organized past a slanted permeable disk. The nanofluid is synthesized by pure water and γ-aluminum oxide nanomaterial under the assumptions that both H_2_O and γ-Al_2_O_3_ are compatible thermally in the absence of no slip between them. Further, solar thermal radiation, convective heat condition and momentum slip are imposed over the flow. The permeability of SRID is subject to $\check{W}>0$ and $\check{W}<0$ against suction and injection of γ-Al_2_O_3_/H_2_O from SRID (Stretching Rotating Inclined Disk). The SRID (Stretching Rotating Inclined Disk) spins about $z=0$ in counter-clockwise orientation. The combined convection influences are also appeared due to the gravity effects on the flow. Moreover, the γ-Al_2_O_3_/H_2_O moves with velocities ($u,v,w$) along headings of increasing ($r,\phi ,z$). The flow scenario of the nanoliquid is portrayed in Fig. 1.

### Empirical nanofluid correlations

The empirical correlations for the nanoliquid are a core ingredient which make it superior than conventional liquids as for as thermal performance concerned. The particular empirical correlations (EC) used in the study are appended as^27,28^:

1 $$ \left( {\widehat{{\rho C_{p} }}} \right)_{{nf}}  = \left( {\widehat{{\rho C_{p} }}} \right)_{{bf}} \left[ {\left( {1 - \phi } \right) + \frac{{\phi \left( {\widehat{{\rho C_{p} }}} \right)_{{np}} }}{{\left( {\widehat{{\rho C_{p} }}} \right)_{{bf}} }}} \right], $$

2 $${\widehat{\rho }}_{nf}=\left[\left(1-\phi \right)+\frac{\phi {\widehat{\rho }}_{np}}{{\widehat{\rho }}_{bf}}\right]{\widehat{\rho }}_{bf},$$

3 $${\left(\widehat{\rho \beta }\right)}_{nf}={\left(\widehat{\rho \beta }\right)}_{bf}\left[\left(1-\phi \right)+\frac{\phi {\left(\widehat{\rho \beta }\right)}_{np}}{{\left(\widehat{\rho \beta }\right)}_{bf}}\right],$$

4 $${\widehat{\mu }}_{nf}={\widehat{\mu }}_{bf}\left(123{\phi }^{2}+7.3\phi +1\right),$$

5 $${\widehat{k}}_{nf}={\widehat{k}}_{bf}\left(4.97{\phi }^{2}+2.72\phi +1\right).$$

In above defined empirical correlations, $\phi , bf, nf, {c}_{p}, \widehat{\rho }, {\widehat{\mu }}_{nf}, {\widehat{k}}_{nf}$ and ${\widehat{k}}_{bf}$ representing the volumetric fraction of γ-nanomaterial, base fluid, nanofluid, heat capacity, thermal conductivity and thermal conductivity of the host liquid, respectively. Further, thermophysical attributes of the (γ-Al_2_O_3_/H_2_O)nf are given in Table 1.

**Table 1:** Thermophysical values of ${\gamma}{\text{Al}}_{2}{\text{O}}_{3}/{\text{H}}_{2}\text{O}$.

Properties	$\widehat{\rho }(\text{kg}/{\text{m}}^{3})$	$\widehat{\beta }\, (1/\text{k})$	${\widehat{c}}_{p}\,(\text{J}/\text{kg K})$	$\widehat{k}\,(\text{W}/\text{mk})$
Pure water (H_2_O)	998.3	20.05 × 10^−5^	4182	$0.60$
Al_2_O_3_	3970	0.85 × 10^−5^	765	$40$

### Development of (γAl_2_O_3_/H_2_O)nf model

According to Boundary Layer (BL) approximation theory, imposed restrictions and empirical correlations, the following model is obtained^4,29^.

#### Conservation of mass, momentum and energy with boundary conditions

6 $$\frac{\partial \tilde{u }}{\partial r}+\frac{\tilde{u }}{\check{r}}+\frac{\partial \tilde{w }}{\partial z}=0,$$

7 $$\tilde{u }\frac{\partial \tilde{u }}{\partial \check{r}}-\frac{\tilde{v }}{{\check{r}}^{2}}+\tilde{w }\frac{\partial \tilde{w }}{\partial z}+\frac{1}{{\rho }_{nf}}\frac{\partial \tilde{P }}{\partial r}-\frac{{\mu }_{nf}}{{\rho }_{nf}}\left\{\frac{{\partial }^{2}\tilde{u }}{\partial {\check{r}}^{2}}+\frac{1}{\check{r}}\frac{\partial \tilde{u }}{\partial \check{r}}-\frac{\tilde{u }}{{\check{r}}^{2}}+\frac{{\partial }^{2}\tilde{w }}{\partial {z}^{2}}\right\}-\frac{{\left(\rho \beta \right)}_{{\rho }_{nf}}}{{\rho }_{nf}}\tilde{g }\left(\tilde{T }-{\tilde{T }}_{\infty }\right)\text{cos}(\check{\psi })+\frac{{\mu }_{nf}}{{\rho }_{nf}}\left(\frac{\tilde{u }}{\stackrel{\sim }{{K}^{*}}}\right)+\tilde{F }{\tilde{u }}^{2}=0,$$

8 $$\tilde{u }\frac{\partial \tilde{v }}{\partial \check{r}}-\frac{\tilde{u }\tilde{v }}{{\check{r}}^{2}}+\tilde{w }\frac{\partial \tilde{v }}{\partial z}-\frac{{\mu }_{nf}}{{\rho }_{nf}}\left\{\frac{{\partial }^{2}\tilde{v }}{\partial {\check{r}}^{2}}+\frac{1}{\check{r}}\frac{\partial \tilde{v }}{\partial \check{r}}-\frac{\tilde{v }}{{\check{r}}^{2}}+\frac{{\partial }^{2}\tilde{v }}{\partial {z}^{2}}\right\}-\frac{{\left(\rho \beta \right)}_{nf}}{{\rho }_{nf}}\tilde{g }\left(\tilde{T }-{\tilde{T }}_{\infty }\right)\text{sin}(\check{\psi })+\frac{{\mu }_{nf}}{{\rho }_{nf}}\left(\frac{\tilde{v }}{\stackrel{\sim }{{K}^{*}}}\right)+\tilde{F }{\tilde{v }}^{2}=0,$$

9 $$\tilde{u }\frac{\partial \tilde{w }}{\partial \check{r}}+\tilde{w }\frac{\partial \tilde{w }}{\partial z}+\frac{1}{{\rho }_{nf}}\frac{\partial \tilde{P }}{\partial z}-\frac{{\mu }_{nf}}{{\rho }_{nf}}\left\{\frac{{\partial }^{2}\tilde{w }}{\partial {\check{r}}^{2}}+\frac{1}{\check{r}}\frac{\partial \tilde{w }}{\partial \check{r}}+\frac{{\partial }^{2}\tilde{w }}{\partial {z}^{2}}\right\}+\frac{{\mu }_{nf}}{{\rho }_{nf}}\left(\frac{\tilde{w }}{\stackrel{\sim }{{K}^{*}}}\right)+\tilde{F }{\tilde{w }}^{2}=0,$$

10 $$\tilde{u }\frac{\partial \tilde{T }}{\partial \check{r}}+\tilde{w }\frac{\partial \tilde{T }}{\partial z}-\frac{{\check{k}}_{nf}}{({\widehat{\rho {C}_{p}})}_{nf}}\left\{\frac{{\partial }^{2}\tilde{T }}{\partial {\check{r}}^{2}}+\frac{1}{\check{r}}\frac{\partial \tilde{T }}{\partial \check{r}}+\frac{{\partial }^{2}\tilde{T }}{\partial {z}^{2}}\right\}-\frac{16{\check{\sigma }}^{*}}{3{k}^{*}({\widehat{\rho {C}_{p}})}_{nf}}\left\{\frac{{\partial }^{2}\tilde{T }}{\partial {\check{r}}^{2}}+\frac{1}{\check{r}}\frac{\partial \tilde{T }}{\partial \check{r}}+\frac{{\partial }^{2}\tilde{T }}{\partial {z}^{2}}\right\}-2\frac{{\mu }_{nf}}{({\widehat{\rho {C}_{p}})}_{nf}}\left\{{\left(\frac{\partial \tilde{u }}{\partial \check{r}}\right)}^{2}+\frac{{\tilde{u }}^{2}}{{\check{r}}^{2}}+{\left(\frac{\partial \tilde{w }}{\partial \check{r}}\right)}^{2}\right\}-\frac{{\mu }_{nf}} {({\widehat{\rho {C}_{p}})}_{nf}} \left \{{\left(\frac{\partial \tilde{v }}{\partial \check{z}}\right)}^{2}+{\left(\frac{\partial \tilde{w }}{\partial \check{r}}+\frac{\partial \tilde{u }}{\partial \check{r}}\right)}^{2}+(\check{r}\frac{\partial }{\partial \check{r}}{\left(\frac{\tilde{v }}{\check{r}}\right)}^{2} \right \} =0.$$

In Eqs. (6)–(10), the velocities are defined as $\tilde{u }, \tilde{v }$ and $\tilde{w }$, and $\tilde{T }$ is the temperature, p is the pressure and g are the gravitational acceleration.

At the surface of SRID (Stretching Rotating Inclined Disk) and ambient location, the fluid moves according to the following rules:

11 $$\left.\begin{array}{c}\tilde{u }=\check{r}\check{S+}{\mu }_{nf}\check{L}\left(\frac{\partial \tilde{u }}{\partial z}\right)\\ \tilde{v }=\check{r}\check{\Omega +}{\mu }_{nf}\check{L}\left(\frac{\partial \tilde{v }}{\partial z}\right), \tilde{w }=-\tilde{W }\\ -{\widehat{k}}_{nf}\left(\frac{\partial \tilde{T }}{\partial z}\right)={\check{h}}_{f}\left({\check{T}}_{f}-\check{T}\right) at z=0\\ \tilde{u }\to 0,\tilde{v }\to 0,\tilde{T }\to {\tilde{T }}_{\infty },\tilde{p }\to {\tilde{p }}_{\infty } \; at \; z\to \infty \end{array}\right\},$$

12 $$\left.\begin{array}{c}\left(\tilde{u },\tilde{v },\tilde{w }\right)=\left(\tilde{r }\check{\Omega }{F}^{{\prime}},\tilde{r }\check{\Omega }{G}^{{\prime}},-{\left(\tilde{r }\check{\Omega }{\nu }_{bf}\right)}^{1/2}F\right)\\ \left(\tilde{p },\tilde{T }\right)=\left({\tilde{p }}_{\infty -}\check{\Omega }{\mu }_{bf}\check{P}, {\check{T}}_{\infty }+\left({\check{T}}-{\check{T}}_{\infty }\right)\beta \right)\\ \eta ={\left(\frac{2\check{\Omega }}{{\nu }_{bf}}\right)}^{1/2}z\end{array}\right\}.$$

The quantities embedded in Eqs. (11) and (12) are stretching/shrinking rate disk is S ($S>0$ and $S<0$ correspond to the stretching and shrinking of RD, respectively), the slip at the wall is $\check{L}$, fluid temperature if ${\check{T}}_{f}$, temperature at ambient location is ${\tilde{T }}_{\infty }$, heat transport coefficient (${\check{h}}_{f}$), free stream pressure (${\tilde{p }}_{\infty }$), permeable velocity ($\tilde{W }$) and rotational velocity ($\check{\Omega }$).

#### Final version of (γAl_2_O_3_-H_2_O)nf

The dimensional physical model reduced into final version after incorporating the empirical (γ-Al_2_O_3_/H_2_O)nf correlations, similarity equations and BC. The version of the model and prescribed (γ-Al_2_O_3_/H_2_O)nf flow conditions are as follows:

13 $$\frac{\left(123{\phi }^{2}+7.3\phi +1\right)}{\left(1-\phi \right)+\frac{\phi {\left(\widehat{\rho {C}_{p}}\right)}_{np}}{{\left(\widehat{\rho {C}_{p}}\right)}_{bf}}}\left(2{F}^{{{\prime\prime\prime}}}-{k}_{1}F{^{\prime}}\right)+2F{F}^{{{\prime\prime}}}-{F}^{{{\prime}}2}+{G}^{2}+\frac{{G}_{r1}}{{\left(Re\right)}^{2}}\left(\frac{\left(1-\phi \right)+\frac{\phi {\left(\widehat{\rho \beta }\right)}_{np}}{{\left(\widehat{\rho \beta }\right)}_{bf}}}{\left(\left(1-\phi \right)+\frac{\phi {\left(\widehat{\rho {C}_{p}}\right)}_{np}}{{\left(\widehat{\rho {C}_{p}}\right)}_{bf}}\right)}\right)\text{cos}\left(\check{\psi }\right)\beta -{F}_{r}{F}^{{{\prime}}2}=0,$$

14 $$\frac{\left(123{\phi }^{2}+7.3\phi +1\right)}{\left(1-\phi \right)+\frac{\phi {\left(\widehat{\rho {C}_{p}}\right)}_{np}}{{\left(\widehat{\rho {C}_{p}}\right)}_{bf}}}\left(2{G}^{{{\prime\prime}}}-{k}_{1}G\right)+2F{G}^{{\prime}}-2{F}^{{\prime}}G-\frac{{G}_{r1}}{{\left(Re\right)}^{2}}\left(\frac{\left(1-\phi \right)+\frac{\phi {\left(\widehat{\rho \beta }\right)}_{np}}{{\left(\widehat{\rho \beta }\right)}_{bf}}}{\left(\left(1-\phi \right)+\frac{\phi {\left(\widehat{\rho {C}_{p}}\right)}_{np}}{{\left(\widehat{\rho {C}_{p}}\right)}_{bf}}\right)}\right)\text{sin}\left(\check{\psi }\right)\beta -{F}_{r}{G}^{2}=0,$$

15 $$\frac{((4.97{\phi }^{2}+2.72\phi +1)+\frac{4}{3}Rd)}{\left(1-\phi \right)+\frac{\phi {\left(\widehat{\rho {C}_{p}}\right)}_{np}}{{\left(\widehat{\rho {C}_{p}}\right)}_{bf}}}{\beta }^{{{\prime\prime}}}+\text{Pr}(F{\beta }^{{\prime}}+\frac{\frac{6Ec}{R{e}^{2}}\left(123{\phi }^{2}+7.3\phi +1\right)}{\left(1-\phi \right)+\frac{\phi {\left(\widehat{\rho {C}_{p}}\right)}_{np}}{{\left(\widehat{\rho {C}_{p}}\right)}_{bf}}}{F}^{{{\prime}}2}+\frac{2Ec\left(123{\phi }^{2}+7.3\phi +1\right)}{\left(1-\phi \right)+\frac{\phi {\left(\widehat{\rho {C}_{p}}\right)}_{np}}{{\left(\widehat{\rho {C}_{p}}\right)}_{bf}}}({F}^{{{\prime\prime}}2}+{G}^{{{\prime}}2})) =0,$$

The appropriate BCs comprising the effects of permeability, thermal and momentum slips are reduced as below:

16 $$\left.\begin{array}{c}{F}^{{\prime}}=\gamma +\delta {\Gamma }_{1}{F}^{{{\prime\prime}}}, G=1+\delta {\Gamma }_{1}{G}^{{\prime}}, F={\Upsilon }_{1}\\ \frac{4.97{\phi }^{2}+2.72\phi +1}{{\check{k}}_{bf}}{\beta }^{{\prime}}=-{B}_{i}\left(1-\beta \right) \, at \; \eta =0\\ {F}^{{\prime}}\to 0,G\to 0,\beta \to 0 \; as \; \eta \to \infty \end{array}\right\}.$$

The governing quantities appeared in the model are summarized in Table 2.

**Table 2:** Physical parameters and their formulas.

S. no.	Symbol	Parameter name	Formula
1	${k}_{1}$	Porosity parameter	${\nu }_{\frac{bf}{\check{\Omega }\stackrel{\sim }{{K}^{*}}}}$
2	$\delta$	Effective dynamic viscosity	$\left(123{\phi }^{2}+7.3\phi +1\right)$
3	$Re$	Reynolds number	$\frac{{\check{r}}\check{\Omega }}{{\nu }_{bf}}$
4	$Rd$	Thermal radiation	$\frac{4{\check{\sigma }}^{*}{\check{T}}_{1}^{3}}{{k}^{*}{\check{k}}_{bf}}$
5	$Ec$	Eckert parameter	$\frac{\check{\Omega }}{{\left({c}_{p}\right)}_{bf}({\check{T}}_{f}-{\check{T}}_{\infty })}$
6	${P}_{r}$	Prandtl number	${\nu }_{\frac{bf}{{\alpha }_{bf}}}$
7	$\gamma$	Stretching parameter	$\frac{\check{S}}{\check{\Omega }}$
8	${\Gamma }_{1}$	Surface slip number	$\check{L}{\mu }_{bf}{\left(\frac{2\check{\Omega }}{{\nu }_{bf}}\right)}^{1/2}$
9	${B}_{i}$	Biot number	$\frac{{h}_{f}}{{\check{k}}_{bf}}{\left({\nu }_{\frac{bf}{2\check{\Omega }}}\right)}^{1/2}$
10	$\Upsilon$	Suction/injection	$\frac{\tilde{W }}{{\left(2\check{\Omega }{\nu }_{bf}\right)}^{1/2}}$
11	${F}_{r}$	Inertia coefficient	$\widehat{r}\tilde{F }$

Further, final version of the quantities that are significant from engineering aspects are the following:

$${\left({R}_{er}\right)}^{0.5}{C}_{F}=\left(123{\phi }^{2}+7.3\phi +1\right)\left\{{\left({F}^{{{\prime\prime}}}\left(\eta =0\right)\right)}^{2}+{\left({G}^{{\prime}}\left(\eta =0\right)\right)}^{2}\right\},$$

$${\left({R}_{er}\right)}^{-0.5}Nu=-\left(\frac{\left(4.97{\phi }^{2}+2.72\phi +1\right)}{{\widehat{k}}_{bf}}+Rd\frac{4}{3}\right){\beta }^{{\prime}}\left(\eta =0\right).$$

### Mathematical analysis of (γAl_2_O_3_-H_2_O)nf

The mathematical analysis of nonlinear governing models cannot be disregarded due to its huge applications in various engineering systems. These are frequently occurred in Solid mechanics, CFD, Fluid mechanics, aerodynamics, GWDA and in mechanical engineering. Therefore, it is imperative to investigate such models mathematically to examine the actual effects of governing parameters. Numerical techniques are best suited for such models. In this regard, RK technique with addition of shooting algorithm is very helpful. This hybrid algorithm primarily works on system of 1st order ODEs. Under consideration (γ-Al_2_O_3_/H_2_O)nf model is extremely nonlinear and coupled. Therefore, firstly the model is reduced into the desired version. Therefore, the following substitutions are decided to acquire the suitable version of the model.

17 $$F={{\check{\mathcal{A}}}_{1}^{*}},{F}^{{\prime}}={{\check{\mathcal{A}}}_{2}^{*}},{F}^{{{\prime\prime}}}={{\check{\mathcal{A}}}_{3}^{*}},{F}^{\prime\prime\prime}={{\check{\mathcal{A}}}^{*\prime}}_{3},G={{\check{\mathcal{B}}}_{4}^{*}},{G}^{{\prime}}={{\check{\mathcal{B}}}_{5}^{*}}, {G}^{{{\prime\prime}}}={{\check{\mathcal{B}}}^{\prime*}_{5}},\beta =\check{{\mathcal{C}}_{7}^{*}}, {\beta }^{{\prime}}={\check{\mathcal{C}}}_{8}^{*},{{\beta }^{{{\prime\prime}}}}={{\check{\mathcal{C}}}^{*\prime}}_{8}.$$

In accordance to these substitutions, the model transformed in the following version:

18 $$\frac{\left(123{\phi }^{2}+7.3\phi +1\right)}{\left(1-\phi \right)+\frac{\phi {\left(\widehat{\rho {C}_{p}}\right)}_{np}}{{\left(\widehat{\rho {C}_{p}}\right)}_{bf}}}\left(2{{\check{\mathcal{A}}}^{*\prime}}_{3}-{k}_{1}{\check{\mathcal{A}}}_{2}^{*}\right)+2{\check{\mathcal{A}}}_{1}^{*}{\check{\mathcal{A}}}_{3}^{*}-{{\check{\mathcal{A}}}_{2}^{*2}}+{{\check{\mathcal{B}}}_{4}^{*2}}+\frac{{G}_{r1}}{{\left(Re\right)}^{2}}\left(\frac{\left(1-\phi \right)+\frac{\phi {\left(\widehat{\rho \beta }\right)}_{np}}{{\left(\widehat{\rho \beta }\right)}_{bf}}}{\left(\left(1-\phi \right)+\frac{\phi {\left(\widehat{\rho {C}_{p}}\right)}_{np}}{{\left(\widehat{\rho {C}_{p}}\right)}_{bf}}\right)}\right)\text{cos}\left(\check{\psi }\right)\check{{\mathcal{C}}_{7}^{*}}-{F}_{r}{{\check{\mathcal{A}}}_{2}^{*2}}=0,$$

19 $$\frac{\left(123{\phi }^{2}+7.3\phi +1\right)}{\left(1-\phi \right)+\frac{\phi {\left(\widehat{\rho {C}_{p}}\right)}_{np}}{{\left(\widehat{\rho {C}_{p}}\right)}_{bf}}}\left(2{{\check{\mathcal{B}}}_{5}^{\prime*}}-{k}_{1}{\check{\mathcal{B}}}_{4}^{*}\right)+2{\check{\mathcal{A}}}_{1}^{*}{\check{\mathcal{B}}}_{5}^{*}-2{\check{\mathcal{A}}}_{2}^{*}{\check{\mathcal{B}}}_{4}^{*}-\frac{{G}_{r1}}{{\left(Re\right)}^{2}}\left(\frac{\left(1-\phi \right)+\frac{\phi {\left(\widehat{\rho \beta }\right)}_{np}}{{\left(\widehat{\rho \beta }\right)}_{bf}}}{\left(\left(1-\phi \right)+\frac{\phi {\left(\widehat{\rho {C}_{p}}\right)}_{np}}{{\left(\widehat{\rho {C}_{p}}\right)}_{bf}}\right)}\right)\text{sin}\left(\check{\psi }\right)\check{{\mathcal{C}}_{7}^{*}}-{F}_{r}{{\check{\mathcal{B}}}_{4}^{*2}}=0,$$

20 $$\frac{\left((4.97{\phi }^{2}+2.72\phi +1)+\frac{4}{3}Rd\right)}{\left(1-\phi \right)+\frac{\phi {\left(\widehat{\rho {C}_{p}}\right)}_{np}}{{\left(\widehat{\rho {C}_{p}}\right)}_{bf}}}{{\check{\mathcal{C}}}_{8}^{*\prime}}+\text{Pr}({\check{\mathcal{A}}}_{1}^{*}{\check{\mathcal{C}}}_{8}^{*}+\frac{\frac{6Ec}{R{e}^{2}}\left(123{\phi }^{2}+7.3\phi +1\right)}{\left(1-\phi \right)+\frac{\phi {\left(\widehat{\rho {C}_{p}}\right)}_{np}}{{\left(\widehat{\rho {C}_{p}}\right)}_{bf}}}{{\check{\mathcal{A}}}_{2}^{*2}}+\frac{2Ec\left(123{\phi }^{2}+7.3\phi +1\right)}{\left(1-\phi \right)+\frac{\phi {\left(\widehat{\rho {C}_{p}}\right)}_{np}}{{\left(\widehat{\rho {C}_{p}}\right)}_{bf}}}({{\check{\mathcal{A}}}_{3}^{*2}}+{{\check{\mathcal{B}}}_{5}^{*2}})) =0.$$

The model is coded in MATHEMATICA 10.0 and then plotted the results against the physical parameters appeared in the model.

## Results with discussion

The Darcy-Forchheimer flow describe the fluid characteristics through porous media and is applicable in the domain of laminar flow. Such flows have extensive applications in the real world. The oil layer underground usually covered by water from downward side or gas cap from above side due to which these fluids mix-up due to permeability and Darcy-Forchheimer Flow (DFF) exist there. These applications comprised in petroleum engineering, seepage of roofs and naturally in rocks.

### $F{^{\prime}}(\eta )$ for (γAl_2_O_3_-H_2_O)nf

**Figure 2:**
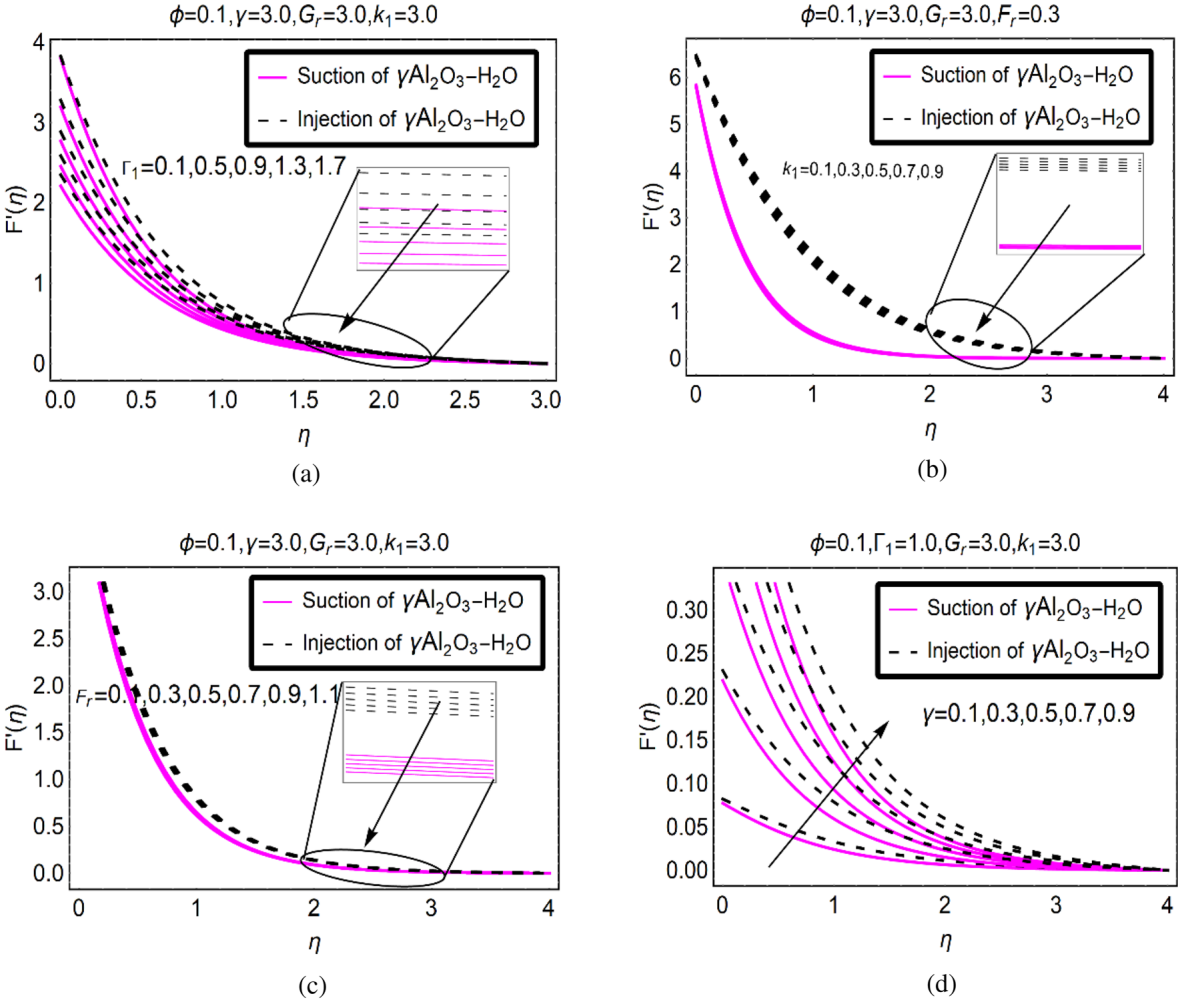
$$F{^{\prime}}(\eta )$$ via (**a**) $${\Gamma }_{1}$$, (**b**) $${k}_{1}$$, (**c**) $${F}_{r}$$ and (**d**) $$\gamma $$.

The surface slip parameter, porosity parameter, ${F}_{r}$ and stretching surface highly affect the motion of (γ-Al_2_O_3_/H_2_O)nf over a SRID (Stretching Rotating Inclined Disk). Therefore, Fig. 2 is decorated to investigate the motion of (γ-Al_2_O_3_/H_2_O)nf for aforementioned parameters, respectively. The results are demonstrated for both suction/injection due to permeability of SRID (Stretching Rotating Inclined Disk). It is inspected that for slip parameter the fluid moves slowly over SRID (Stretching Rotating Inclined Disk). In the vicinity of the surface, these effects are very strong and gradually slow down towards ambient location of the surface. For suction of (γ-Al_2_O_3_/H_2_O)nf, the velocity abruptly drops. Physically, more fluid particles stuck with the surface of SRID due to which the particles of successive fluid layers move slowly and the particles momentum drops. While, for injection of (γ-Al_2_O_3_/H_2_O)nf, the fluid particles move quite abrupt than suction case. The physical aspects of this behaviour are that the injecting fluid give quite extra momentum to the particles due to which the momentum drops quite slowly. In similar pattern, the fluid motion reduces for ${k}_{1}$ and ${F}_{r}$, respectively. These trends are decorated in Fig. 2a–c, respectively.

A very interesting behavior of $\gamma$ on the motion of (γ-Al_2_O_3_/H_2_O)nf is depicted in Fig. 2d. The stretching of the surface is beneficial for the motion over SRID (Stretching Rotating Inclined Disk). At the surface, the fluid moves very abruptly due to increasing $\gamma$. Physically, the flowing area intensifies due to the stretching of the surface and fluid particles flow freely over it which ultimately increases the motion. For injecting fluid, the motion behaviour is very prominent than suction because the fluid particles drag at the surface due to suction which resists the motion. These are portrayed in Fig. 2d.

The study of the dynamics of nanoliquids over a rotating disk broadly used and very popular in electrochemical engineering, brakes, gears and in gas turbine engines. Therefore, alterations in the physical constraints significantly affect the dynamics of the fluid which are explained above against the Fig. 2a–d, respectively. It imperative from engineering point of view to use a disk with pores at the slippery disk surface and stretching effects. One of the advantages of these physical situations is the purification of various industrial products that needs slow motion of the fluid in the flowing region. If aforementioned effects are integrated and imposed over the desired region then it controls the fluid motion due to which the impurities settle down at the bottom and purified products could be achieved.

### $G(\eta )$ for (γAl_2_O_3_-H_2_O)nf

**Figure 3:**
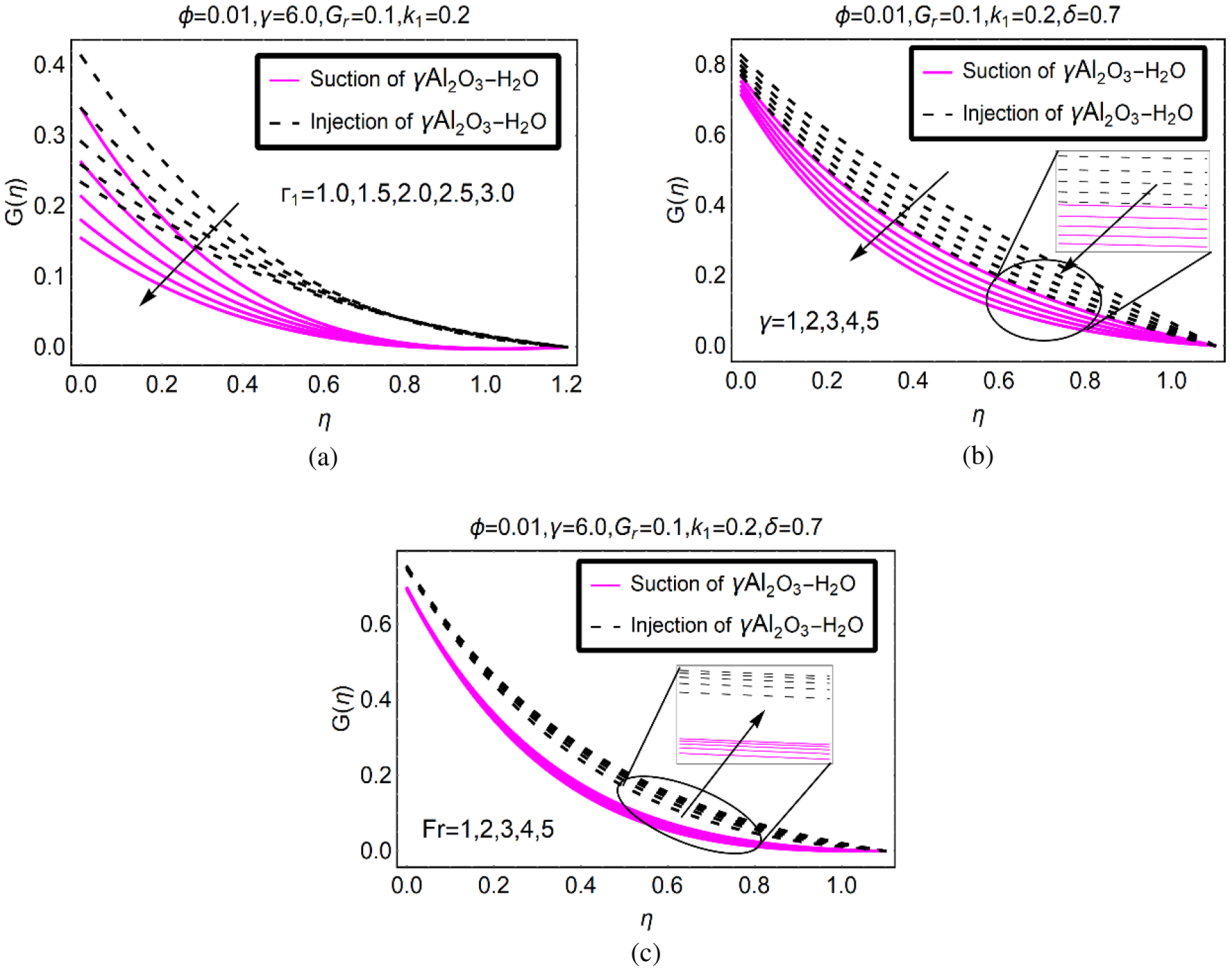
$$G(\eta )$$ via (**a**) $${\Gamma }_{1}$$, (**b**) $$\gamma $$, (**c**) $${F}_{r}$$.

The velocity trends $G(\eta )$ for higher slip, stretching and ${F}_{r}$ over the desired domain are pictured in Fig. 3, respectively. The stronger slip effects resists the rotational motion of (γ-Al_2_O_3_/H_2_O)nf in Fig. 3a. The fluid moves very slowly at the surface and maximum decreasing trends are examined for suction of (γ-Al_2_O_3_/H_2_O)nf. On the other side, the velocity drops quite slowly for injecting fluid. Further, the motion of the fluid vanishes at ambient location which gratify the imposed flow conditions. The stretching rate of the surface highly affects the rotational motion of (γ-Al_2_O_3_/H_2_O)nf in the existence of combined convection effects. These effects are noticed from Fig. 3b. The motion of (γ-Al_2_O_3_/H_2_O)nf rises by inducing DF effects in the governing model. These are elaborated in Fig. 3c. From this it is clear that DF is helpful to intensifies the motion of (γ-Al_2_O_3_/H_2_O)nf over SRID (Stretching Rotating Inclined Disk). The maximum rise in the rotational motion is observed for injection case.

### Thermal behavior of (γAl_2_O_3_/H_2_O)nf

**Figure 4:**
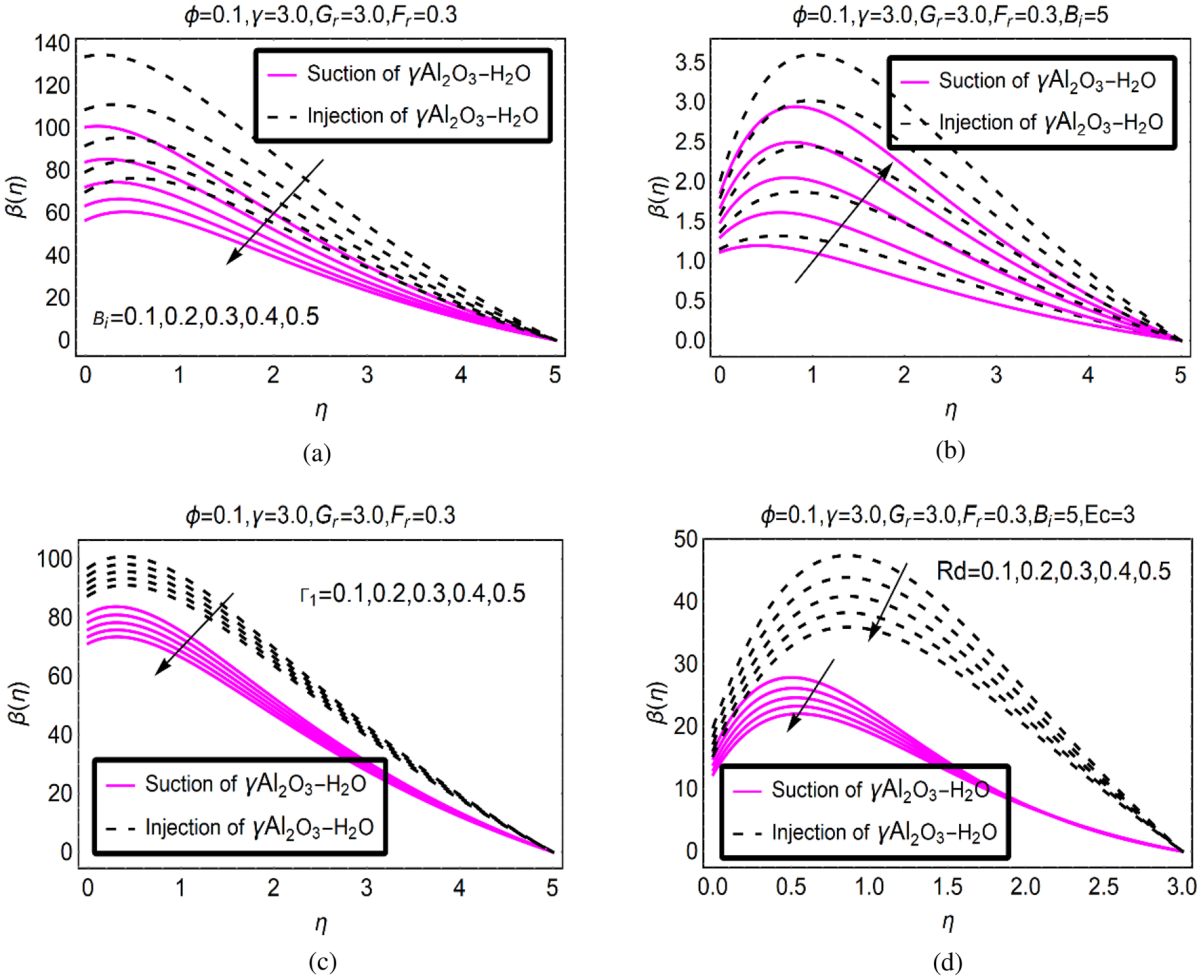
$$\beta (\eta )$$ via (**a**) $${\text{B}}_{i}$$, (**b**) $$Ec$$, (**c**) $${\Gamma }_{1}$$ and (**d**) $$Rd$$.

Figure 4 is organized to analyze the temperature trends in (γ-Al_2_O_3_/H_2_O)nf over radiative SRID (Stretching Rotating Inclined Disk). From the analysis of the plotted results (Fig. 4a–d), it is examined that the temperature declines for (γ-Al_2_O_3_/H_2_O)nf under emerging governing quantities. The rapid decrement is noticed from Fig. 4a and 4d while; quite slow drops in the temperature is observed for higher ${\Gamma }_{1}$ in (γ-Al_2_O_3_/H_2_O)nf. Usually, the temperature rises due to surface convection and thermal radiation however; in this study the influences of DF are induced in the model which significantly affect the temperature of (γ-Al_2_O_3_/H_2_O)nf. The viscous dissipation playing vital role in the temperature enhancement in (γ-Al_2_O_3_/H_2_O)nf. For more dissipative (γ-Al_2_O_3_/H_2_O)nf, the temperature intensifies abruptly. Physically, it intensifies the internal energy between the fluid particles which favors the temperature.

### Thermophysical featuring in (γAl_2_O_3_/H_2_O)nf

**Figure 5:**
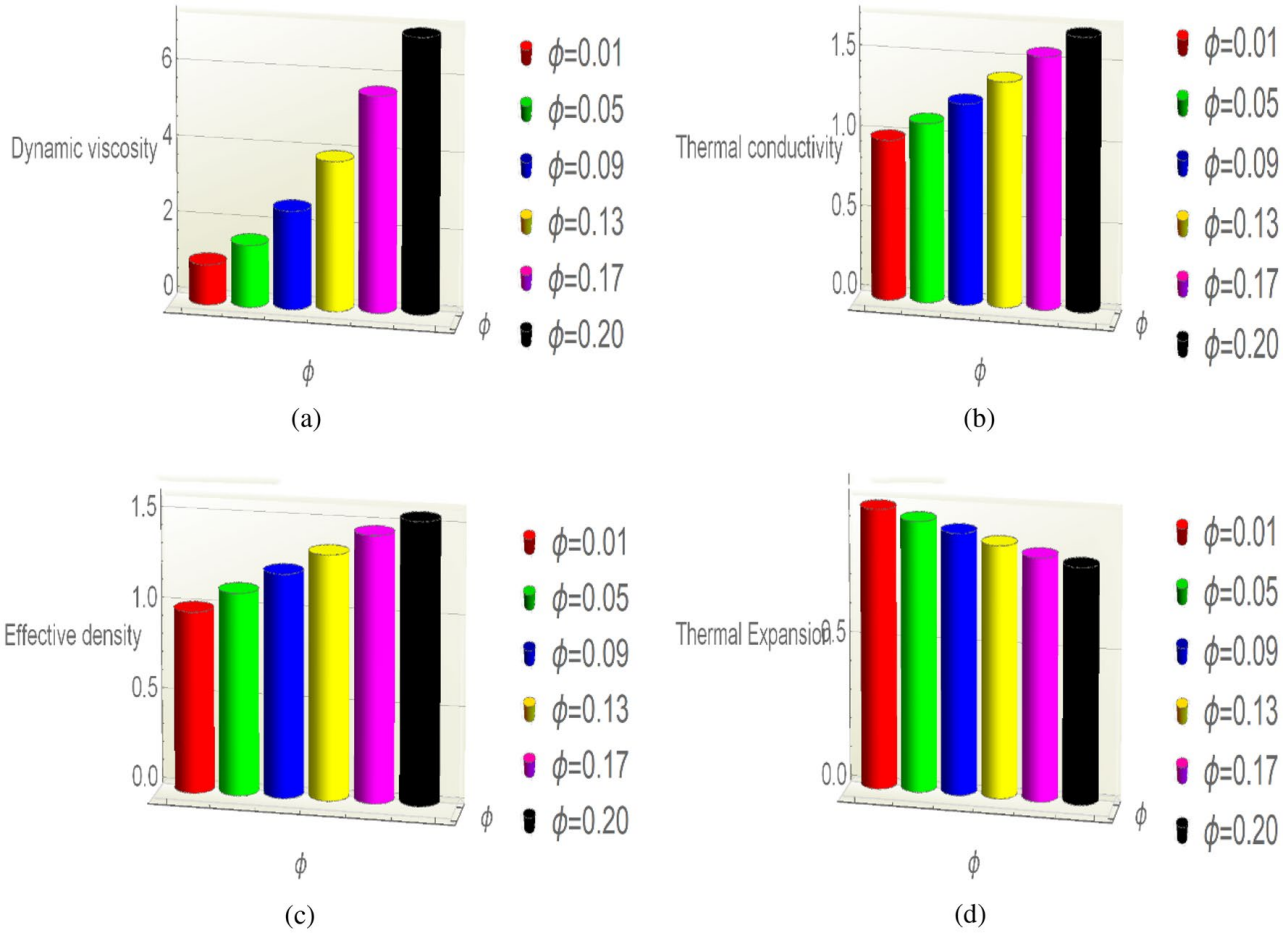
Thermophysical featuring (**a**) dynamic viscosity, (**b**) thermal conductivity, (**c**) effective density, (**d**) thermal expansion via $\phi$.

This subsection is designed to observe the behaviour of thermophysical featuring of (γ-Al_2_O_3_/H_2_O)nf by intensifying the fraction factor of the nanomaterial. Thermophysical attributes of the nanofluids are very important for the analysis of nanofluids. Therefore, Fig. 5 is organized for said purpose. From this, it is concluded that the fraction factor dynamic viscosity, thermal conductance and effective density enhances due to higher fraction factor. While; thermal expansion of (γ-Al_2_O_3_/H_2_O)nf drops which knowingly disturb the temperature behaviour.

### Quantities of engineering scope

**Figure 6:**
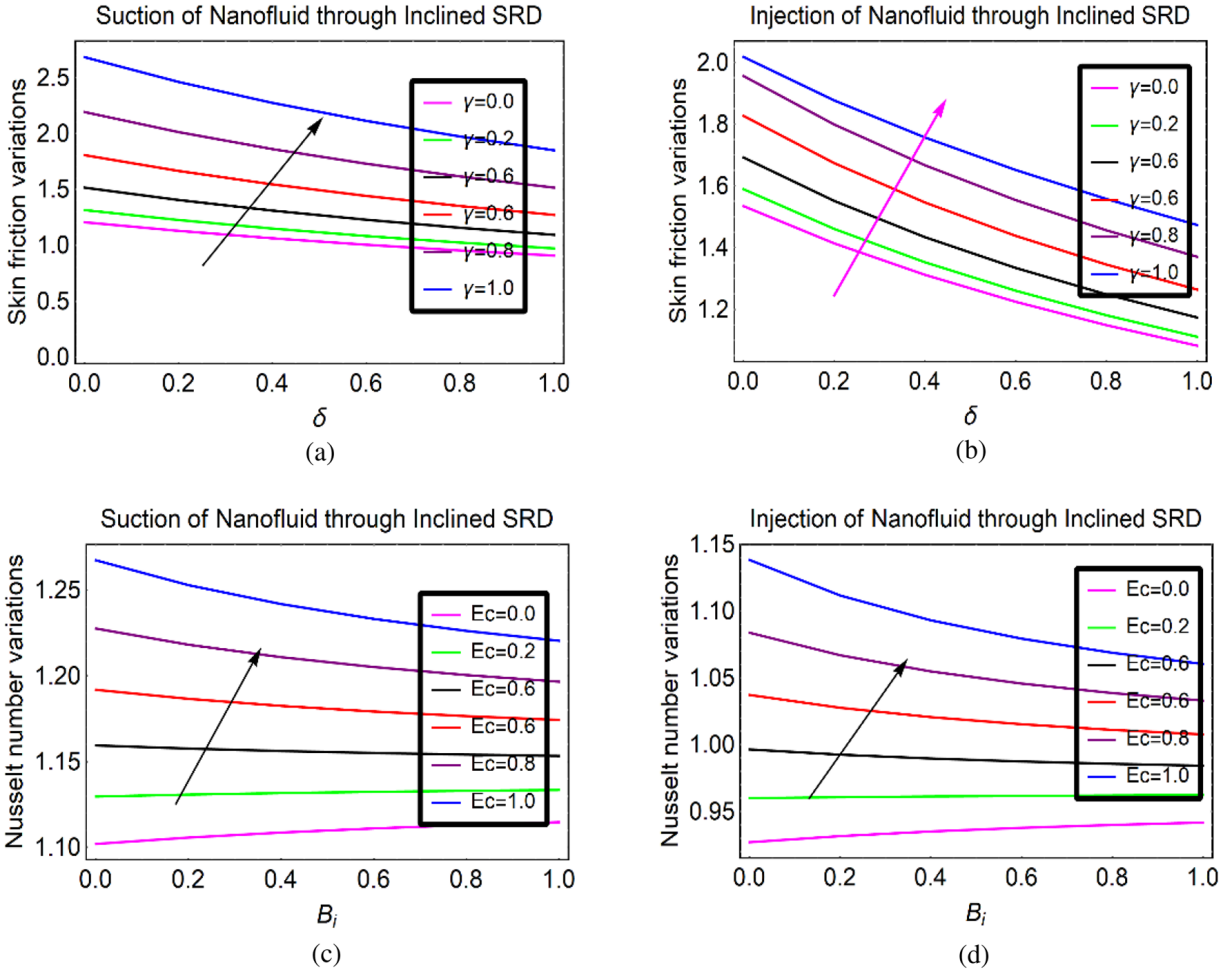
Shear stresses (**a,b**) and local heat tranposrt rate (**c,d**) via $\gamma$ and $Ec$, respectiveyl.

The changes in walls shear stresses and local thermal rate analysis in the nanofluids is imperative for various industrial and engineering purposes. This subsection is providing the behaviour of aforementioned quantities under stretching ration and viscous dissipation, respectively. The shear stresses rapidly move on the surface due to stretching of SRID (Stretching Rotating Inclined Disk) by altering $\gamma$. Similarly, viscus dissipation is a significant phenomenon to enhance the internal energy of (γ-Al_2_O_3_/H_2_O)nf which ultimately make it good heat conductor and prolong their applications in industries and engineering systems. These trends are pictured in Fig. 6.

## Concluding remarks

The Darcy–Forchheimer Flow (DFF) of (γ-Al_2_O_3_/H_2_O)nf by inducing the novel effects of thermal radiation, velocity slip and convective heat condition over a SRID is organized. The final version of the model is attained via empirical correlations of (γ-Al_2_O_3_/H_2_O)nf and similarity equations. After that, a deep discussion for the flow regimes provided against embedded physical constraints. It is revealed that:

- The (γ-Al_2_O_3_/H_2_O)nf motion abruptly rises due to the stretching effects at the surface and these vanishes asymptotically at ambient location from the surface.
- The inertia coefficient F_r_ resists the fluid motion ($F{^{\prime}}(\eta )$) and it favors the tangential fluid velocity $G(\eta )$.
- The presence of pores at the surface (porosity parameter ${k}_{1}$) lead to decrement in the motion of (γ-Al_2_O_3_/H_2_O)nf.
- The temperature of DFF declines against the physical constraint; therefore, it would be beneficial for cooling applications in various industries.
- The shear drags at the surface increases due to suction and injection of (γ-Al_2_O_3_/H_2_O)nf in the presence of varying $\gamma$.
- The rate of heat transport significantly enhanced due to convectively heated disk and viscous dissipation.
- The volumetric fraction played vital role in the enhancement of thermophysical correlations of (γ-Al_2_O_3_/H_2_O)nf.

The used nanoparticles significantly applicable in mechanical engineering and aerodynamics. As, the study reveals that these fluids have good coolant characteristics, these extensively used in coolant engine of vehicle, aircraft, missile technology and combustion channel walls of fighter jet etc.

## Data Availability

The authors declared no additional data for this manuscript.

## Nomenclature

($\tilde{u},\tilde{v},\tilde{w}$): Velocity components along coordinates
SRID: Stretching rotating inclined disk
SRD: Stretching rotating disk
DFF: Darcy–Forchheimer flow
BL: Boundary layer
BC’s: Boundary conditions
EC: Empirical correlations
$$\tilde{g}$$: Gravitational acceleration ($${\text{ms}}^{ - 2}$$)
$$\tilde{T}$$: Temperature (K)
$\tilde{T}_{\infty }$: Temperature at ambient location (K)
$$\tilde{P}$$: Pressure (Pa)
$\tilde{p}_{\infty }$: Temperature at ambient location (Pa)
$$\hat{\rho }_{nf}$$: Effective density ($${\text{kg}}/{\text{m}}^{3}$$)
$\hat{\rho }_{np}$: Density of nanoparticles (${\text{kg}}/{\text{m}}^{3}$)
$\hat{\rho }_{bf}$: Density of water (${\text{kg}}/{\text{m}}^{3}$)
$\hat{\mu }_{nf}$: Effective dynamic viscosity (${\text{kg}}/{\text{ms}}$)
$\hat{\mu }_{bf}$: Effective dynamic of base fluid (${\text{kg}}/{\text{ms}}$)
$\hat{\beta }_{np}$: Nanoparticle thermal expansion (K^−1^)
$\hat{\beta }_{bf}$: Base liquid thermal expansion (K^−1^)
$\left( {\widehat{{\rho C_{p} }}} \right)_{nf}$: Nanoliquid effective heat capacitance (${\text{Jkg}}^{2} {\text{m}}^{3} /{\text{K}}$)
$\left( {\widehat{{\rho C_{p} }}} \right)_{bf}$: Base liquid heat capacitance (${\text{Jkg}}^{2} {\text{m}}^{3} /{\text{K}}$)
$\left( {\widehat{{\rho C_{p} }}} \right)_{np}$: Nanoparticles heat capacitance (${\text{Jkg}}^{2} {\text{m}}^{3} /{\text{K}}$)
$\hat{k}_{nf}$: Effective thermal conductivity of nf (${\text{W}}/{\text{mK}}$)
$\hat{k}_{np}$: Thermal conductivity of np (${\text{W}}/{\text{mK}}$)
$\hat{k}_{bf}$: Thermal conductivity of bf (${\text{W}}/{\text{mK}}$)
$\check{h}_{f}$: Coefficient of heat transport (${\text{W}}/{\text{m}}^{2} {\text{K}}$)
$\phi$: Volumetric fraction factor
$$k_{1}$$: Porosity parameter
$G_{r}$: Thermal Grashof number
$Re$: Local Reynold number
$Rd$: Thermal radiation number
$$Ec$$: Eckert number
$$P_{r}$$: Prandtl number
$$\gamma$$: Stretching number
${\Gamma }_{1}$: Surface slip number
$$B_{i}$$: Biot number
${\Upsilon }$: Suction/injection number
$$F_{r}$$: Inertia coefficient
$C_{F}$: Coefficient of skin friction
$N_{u}$: Local Nusselt number

## References

[1] Choi SUS (1995). Enhancing thermal conductivity of fluids with nanoparticles. ASME.

[2] Kumar B, Seth GS, Singh MK, Chamkha AJ (2021). Carbon nanotubes (CNTs)-based flow between two spinning discs with porous medium, Cattaneo-Christov (non-Fourier) model and convective thermal condition. J. Therm. Anal. Calorim..

[3] Li YX, Muhammad T, Bilal M, Khan MA, Ahmadian A, Pansera BA (2021). Fractional simulation for Darcy–Forchheimer hybrid nanoliquid flow with partial slip over a spinning disk. Alex. Eng. J..

[4] Rout H, Mohapatra SS, Shaw S, Muhammad T, Nayak MK, Makinde OD (2021). Entropy optimization for Darcy-Forchheimer electro-magneto-hydrodynamic slip flow of ferronanofluid due to stretching/shrinking rotating disk. Waves Random Complex Media..

[5] Turkyilmazoglu M (2014). Nanofluid flow and heat transfer due to a rotating disk. Comput. Fluids.

[6] Zangooee MR, Hosseinzadeh KH, Ganji DD (2019). Hydrothermal analysis of MHD nanofluid (TiO2-GO) flow between two radiative stretchable rotating disks using AGM. Case Stud. Therm. Eng..

[7] Tassaddiq A, Khan S, Bilal M, Gul T, Mukhtar S, Shah Z, Bonyah E (2020). Heat and mass transfer together with hybrid nanofluid flow over a rotating disk. AIP Adv..

[8] Gul T, Usman M, Khan I, Nasir S, Saeed A, Khan A, Ishaq M (2021). Magneto hydrodynamic and dissipated nanofluid flow over an unsteady turning disk. Adv. Mech. Eng..

[9] Asma A, Othman WAM, Muhammad T, Mallawi F, Wong BR (2019). Numerical study for magnetohydrodynamic flow of nanofluid due to a rotating disk with binary chemical reaction and Arrhenius activation energy. Symmetry..

[10] Adnan, Zaidi SZA, Khan U, Abdeljawad T, Ahmed N, Mohyud-Din ST, Khan I, Nisar KS (2020). Investigation of thermal transport in multi-shaped Cu nanomaterial-based nanofluids. Materials.

[11] Ahmed N, Abbasi A, Saba F, Khan U, Mohyud-Din ST (2018). Flow of ferro-magnetic nanoparticles in a rotating system: A numerical investigation of particle shapes. Indian J. Phys..

[12] Adnan, Khan SIU, Khan U, Ahmed N, Mohyud-Din ST, Nisar KS (2021). Thermal transport investigation in AA7072 and AA7075 aluminum alloys nanomaterials based radiative nanofluids by considering the multiple physical flow conditions. Sci. Rep..

[13] Mandal PK, Seth GS, Sarkar S, Chamkha A (2021). A numerical simulation of mixed convective and arbitrarily oblique radiative stagnation point slip flow of a CNT-water MHD nanofluid. J. Therm. Anal. Calorim..

[14] Bhattacharyya A, Seth GS, Kumar R, Chamkha A (2020). Simulation of Cattaneo-Christov heat flux on the flow of single and multi-walled carbon nanotubes between two stretchable coaxial rotating disks. J. Therm. Anal. Calorim..

[15] Kumar R, Bhattacharyya A, Seth GS, Chamkha AJ (2021). Transportation of magnetite nanofluid flow and heat transfer over a rotating porous disk with Arrhenius activation energy: Fourth order Noumerov’s method. Chin. J. Phys..

[16] Adnan A, Khan U, Ahmed N, Mohyud-Din ST (2021). Enhanced heat transfer in H2O inspired by Al2O3 and γAl2O3 nanomaterials and effective nanofluid models. Adv. Mech. Eng..

[17] Seth GS, Kumar RS, Bhattacharyya A (2018). Entropy generation of dissipative flow of carbon nanotubes in rotating frame with Darcy-Forchheimer porous medium: A numerical study. J. Mol. Liq..

[18] Seth GS, Tripathi R, Mishra MK (2017). Hydromagnetic thin film flow of Casson fluid in non-Darcy porous medium with Joule dissipation and Navier’s partial slip. Appl. Math. Mech..

[19] Kumar R, Seth GS, Bhattacharyya A (2019). Entropy generation of von Karman's radiative flow with Al2O3 and Cu nanoparticles between two coaxial rotating disks: A finite-element analysis. Eur. Phys. J. Plus.

[20] Adnan, Khan U, Ahmed N, Mohyud-Din ST, Alharbi SO, Khan I (2022). Thermal improvement in magnetized nanofluid for multiple shapes nanoparticles over radiative rotating disk. Alexand. Eng. J..

[21] Nandi S, Kumbhakar B, Seth GS, Chamkha AJ (2021). Features of 3D magneto-convective nonlinear radiative Williamson nanofluid flow with activation energy, multiple slips and Hall effect. Phys. Scripta.

[22] Li YX, Muhammad T, Bilal M, Khan M, Ahmadian A, Pansera BA (2021). Fractional simulation for Darcy–Forchheimer hybrid nanoliquid flow with partial slip over a spinning disk. Alexand. Eng. J..

[23] Xu YJ, Bilal M, Al-Mdallal Q, Khan MA, Muhammad T (2021). Gyrotactic micro-organism flow of Maxwell nanofluid between two parallel plates. Sci. Rep..

[24] Adnan, Khan U, Ahmed N, Mohyud-Din ST, Hamadneh NN, Khan I, Andualem M (2021). The dynamics of H2O suspended by multiple shaped Cu nanoadditives in rotating system. J. Nanomater..

[25] Lv YP, Algehyne EA, Alshehri MG, Alzahrani E, Bilal M (2021). Numerical approach towards gyrotactic microorganisms hybrid nanoliquid flow with the hall current and magnetic field over a spinning disk. Sci. Rep..

[26] Ahmadian A, Bilal M, Khan MA, Asjad MI (2020). Numerical analysis of thermal conductive hybrid nanofluid flow over the surface of a wavy spinning disk. Sci. Rep..

[27] Rashidi MM, Ganesh NV, Hakeem AKA, Ganga B, Lorenzini G (2016). Influences of an effective Prandtl number model on nano boundary layer flow of c Al2O3–H2O and c Al2O3–C2H6O2 over a vertical stretching sheet. Int. J. Heat Mass Transf..

[28] Khan U, Adnan, Ahmed N, Mohyud-Din ST (2020). Surface thermal investigation in water functionalized Al2O3 and γAl2O3 nanomaterials-based nanofluid over a sensor surface. Appl. Nanosci..

[29] Shaw S, Samantaray SS, Misra A, Nayak MK, Makinde OD (2022). Hydromagnetic flow and thermal interpretations of Cross hybrid nanofluid influenced by linear, nonlinear and quadratic thermal radiations for any Prandtl number. Int. Commun. Heat Mass Transf..

